# An analysis of common foodborne parasitic zoonoses in slaughtered sheep and cattle in Tehran, Iran, during 2015-2018

**DOI:** 10.14202/vetworld.2018.1486-1490

**Published:** 2018-10-24

**Authors:** Ali Pezeshki, Hadi Aminfar, Majid Aminzare

**Affiliations:** 1 Department of Parasitology and Mycology, School of Medicine, Zanjan University of Medical Sciences, Zanjan, Iran; 2 Ph.D. Candidate of Internal Medicine of Large Animals, Faculty of Veterinary Medicine, Urmia University, Urmia, Iran; 3 Department of Food Safety and Hygiene, School of Public Health, Zanjan University of Medical Sciences, Zanjan, Iran

**Keywords:** dicrocoeliasis, fascioliasis, hydatidosis, sheep and cattle, slaughterhouse

## Abstract

**Background and Aim:**

Cystic echinococcosis, *Echinococcus granulosus*, and liver flukes, such as *Fasciola* spp. and *Dicrocoelium dendriticum*, are important parasitic zoonoses, where they able to cause significant veterinary, medical, and economic problems. The present study was carried out to obtain the updated knowledge on the frequency of hydatidosis, fasciolosis, and dicrocoeliosis in the slaughtered sheep and cattle.

**Materials and Methods:**

Information were collected from meat inspection records using systematically visual inspection, palpation, and incision of the visceral organs in the industrial abattoir in Tehran, the capital of Iran, between February 1, 2015, and January 31, 2018. For an analysis of the data, SPSS version 16 was applied.

**Results:**

The hydatidosis infection in sheep and cattle was 2.48% and 2.25%, respectively. With respect to liver flukes, 0.62% and 0.25% sheep and cattle were infected by *Fasciola* spp., respectively; furthermore, 2.86% sheep and 0.79% cattle were positive for *D. dendriticum*.

**Conclusion:**

The findings will provide considerable awareness for the future monitoring and control of these potentially important infections.

## Introduction

Cystic echinococcosis or hydatidosis, fasciolosis, and dicrocoeliosis are common foodborne parasitic infections worldwide which caused by *Echinococcus granulosus*, *Fasciola* spp., and *Dicrocoelium dendriticum*, respectively [1-4]. *E. granulosus* is a small cestode belonging to the Taeniidae family [5]. In the life cycle of this parasite, dogs and other canids are definitive hosts and ruminants as well as human are intermediate hosts in which hydatid cyst occurs [6]. Cystic echinococcosis is endemic in Iran [2,7]. *Fasciola* spp. and *D. dendriticum* are common liver flukes [8]. However, these parasites are considered as the important parasites of domestic and wild ruminants such as sheep, cattle, goat, camel, and deer. In addition, human is identified as an accidental host for the mentioned parasites, sporadically. [9-11]. Several investigations showed that *Fasciola* spp. and *D. dendriticum* are widespread in various parts of Iran [12-14]. *E. granulosus* and liver flukes inflict not only significant medical problems in humans but also cause enormous economic losses in animals due to the mortality, organ condemnation, and decreasing of the meat, milk, and wool production [11,15].

The abattoir data are a useful tool for the study of epidemiological aspects of specific infections, evaluation of disease status which has no other suitable diagnostic techniques, screening livestock products with abnormalities that are hazardous for human use, and estimating the financial damages of affected organ condemnation [16-18].

The incidence of *E. granulosus*, *Fasciola* spp., and *D. dendriticum* has been determined in the slaughtered livestock in some parts of Iran [11,19-23], but there is not enough information from Tehran Province, Iran. The present study was designed to measure the frequency rate of common foodborne parasitic diseases, hydatidosis, fasciolosis, and dicrocoeliosis, in the slaughtered sheep and cattle for the period of February 1, 2015-January 31, 2018.

## Materials and Methods

### Ethical approval

Samples were collected from slaughtered animals from slaughterhouses in Tehran. No animals were slaughtered for the study. However, authors used retrospective data for this study.

### Sampling

A retrospective survey was carried out for 3 years, from February 1, 2015, to January 31, 2018, to investigate the frequency of hydatid cyst and liver flukes, *Fasciola* spp. and *D. dendriticum*, in the slaughtered sheep and cattle in Tehran, the capital city of Iran, which is located between the latitudes 35° 40’ N and longitudes 5° 19’ E. Tehran is the most populous city in Iran and Western Asia with a population of around 8.8 million in the city and 15 million in the larger metropolitan area of greater Tehran. The climate of the city is mild in spring and autumn, hot and dry in summer, and cold and wet in winter with an average annual rainfall of about 245 mm. The mean yearly humidity is approximately 43%. A total of 571,991 sheep and 80,001 cattle were evaluated considering liver and lung in an industrial slaughterhouse in Tehran Province during 36 months period, from February 1, 2015, to January 31, 2018. The data on parasites were obtained daily using observation, palpation, and incisions of the carcasses and visceral organs particularly the liver and lung according to the standards of the Iranian Veterinary Organization. Based on the morphological characteristics, the parasites were indicated as hydatid cyst, *Fasciola* spp., and *D. dendriticum*. The frequency rate was sorted on season basis.

### Statistical analysis

SPSS software Version 16 (SPSS Inc., Chicago, IL, USA) was used for analyzing data. One-way ANOVA and Chi-square tests were used to determine contamination abundance and seasonal prevalence correlation, respectively.

## Results

From 571,991 sheep and 80,001 cattle slaughtered in the studied abattoir from February 1, 2015, to January 31, 2018, 14,221 (2.48%) and 1811 (2.25%) were positive for hydatidosis, respectively. Infection of the liver and lung due to the hydatidosis for sheep was 1.17% and 1.31%, respectively, while the corresponding infections for cattle were 0.93% and 1.32%, respectively. With respect to liver flukes, 0.62% sheep and 0.25% cattle were infected by *Fasciola* spp. Moreover, *D. dendriticum* was detected in 2.86% sheep and 0.79% cattle, respectively (Table-1). The frequency of hydatid cyst, *Fasciola* spp., and *D. dendriticum* in the current investigation during four seasons is presented in Table-2.

**Table-1:** Annual frequency rate of hydatidosis, fasciolosis, and dicrocoeliosis in the slaughtered sheep and cattle in Tehran, Iran, February 1, 2015-January 31, 2018.

Year	Sheep					Cattle				
	
	Number slaughtered	Hydatidosis (liver) (%)	Hydatidosis (lung) (%)	Dicrocoeliosis (%)	Fasciolosis (%)	Number slaughtered	Hydatidosis (liver) (%)	Hydatidosis (lung) (%)	Dicrocoeliosis (%)	Fasciolosis (%)
2015-2016	206325	2306 (1.11)	2373 (1.15)	5741 (2.78)	1196 (0.57)	28372	246 (0.86)	422 (1.48)	299 (1.05)	86 (0.30)
2016-2017	216003	2450 (1.13)	2881 (1.33)	6032 (2.79)	1355 (0.62)	26254	271 (1.03)	327 (1.24)	172 (0.65)	56 (0.21)
2017-2018	149663	1940 (1.29)	2271 (1.51)	4607 (3.07)	1048 (0.70)	25375	230 (0.90)	315 (1.24)	167 (0.65)	62 (0.24)
Total	571991	6696 (1.17)	7525 (1.31)	16380 (2.86)	3599 (0.62)	80001	747 (0.93)	1064 (1.32)	638 (0.79)	204 (0.25)

**Table-2:** Seasonal frequency of hydatidosis, fasciolosis, and dicrocoeliosis in the slaughtered sheep and cattle in Tehran, Iran, February 1, 2015-January 31, 2018.

Animal species	Spring	Summer	Autumn	Winter	p-value
Sheep					
Animal slaughtered	167743	140557	111287	152404	
Hydatidosis (liver) (%)	1718 (1.02)	1503 (1.07)	1291 (1.16)	2184 (1.43)	<0.001
Hydatidosis (lung) (%)	2074 (1.23)	2218 (1.57)	1477 (1.32)	1756 (1.15)	<0.001
Dicrocoeliosis (%)	3988 (2.37)	3467 (2.46)	3768 (3.38)	5157 (3.38)	<0.001
Fasciolosis (%)	852 (0.50)	741 (0.52)	816 (0.73)	1190 (0.78)	<0.001
Cattle					
Animal slaughtered	19602	19198	19818	21383	
Hydatidosis (liver) (%)	205 (1.04)	210 (1.09)	148 (0.74)	184 (0.86)	0.0008
Hydatidosis (lung) (%)	307 (1.56)	308 (1.60)	221 (1.11)	228 (1.06)	<0.001
Dicrocoeliosis (%)	198 (1.01)	175 (0.91)	114 (0.57)	151 (0.70)	<0.001
Fasciolosis (%)	53 (0.27)	36 (0.18)	43 (0.21)	72 (0.33)	0.0157

## Discussion

*E. granulosus*, *Fasciola* spp., and *D. dendriticum* are significant helminthic zoonoses [23,24]. These parasites have considerable medical, veterinary, and economic complication worldwide [11,22,25]. Iran is an important endemic focus of hydatidosis and liver flukes [8,12,13,23,26-28]. Various studies on cystic echinococcosis, which have been carried out in Iran, showed that the infection is generally found in sheep, cattle, goats, camels, and human [2,29-33]. Dogs, wolves, and jackals are definitive host of *E. granulosus*. Moreover, investigations have documented the presence of fasciolosis and dicrocoeliosis among sheep, cattle, goats, and humans throughout the country [9-11].

There has been a positive relationship between the frequency of these parasites in animals and the possibility of human infection [34]. Therefore, this alarming frequency of *E. granulosus*, *Fasciola* spp., and *D. dendriticum* in animals represents a potential hazard for human beings [23,25]. Based on the literature, the prevalence of fasciolosis, dicrocoeliosis, hydatidosis of sheep, and hydatidosis of cattle in various parts of Iran ranged from 2.3% to 5.5%, 2.5% to 4.8%, 1% to 27.5%, and 1% to 28.5%, respectively [32,35]. Studies have documented that the presence of these parasitic zoonoses reduces in Central and South Provinces of Iran because of the few intermediate hosts, lack of livestock population, and intense environment [22,36]. In the present study, mean contamination of hydatidosis in sheep and cattle was 2.48% and 2.25%, respectively.

On the other hand, the mean frequency of dicrocoeliasis and fasciolosis was 2.86% and 0.62% in sheep and 0.79% and 0.25% in cattle, respectively. Based on inspection of the internal organs from abattoirs, the infection rate of hydatidosis was as in Kashan, Iran (sheep 2.25% and cattle 4%), Ardabil, Iran (sheep 74.4% and cattle 38.3%), Khorasan, Iran (sheep 2.65% and cattle 4.15%), Lorestan, Iran (sheep 21.22% and cattle 26.71%), Shiraz, Iran (sheep 45.52% and cattle 11.6%), Torbat-E-Heidarieh, Iran (sheep 7.95% and cattle 8.05%), and Kermanshah, Iran (sheep 3% and cattle 7.65%) [11,19,22,27,37-39]. The differences in frequency of hydatid cyst in various investigations are due to culture, geographical parameters, climate changes, environmental conditions, livestock husbandry, type of population of final hosts and sample collection [11,23,25]. Various frequencies of fasciolosis have previously been reported from the different parts of Iran, including 5.3% in sheep and 25.9% in cattle in Ardabil, 2.49% in sheep and 1.86% in cattle in Shiraz, 1.93% in sheep and 1.89% in cattle in Torbat-E-Heidarieh, and 1.23% in sheep and 5.99% in cattle in Kermanshah [11,22,23,27], and the frequency rate of dicrocoeliosis regarding abattoirs data in the different regions of Iran is as follows: Ardabil (sheep 6.8% and cattle 10.6%), Shiraz (sheep 0.026% and cattle 0.91%), Torbat-E-Heidarieh (sheep 1.97% and cattle 2.16%), and Kermanshah (sheep 2.33% and cattle 4.81%) [11,22,23,27]. The existence of various levels of liver flukes, *Fasciola* spp., and *D. dendriticum* infections could be related to the environmental factors, the weather conditions, the ecological parameters, the livestock management system, and the sample collection [11,14]. In the current survey, the infection rates of *Fasciola* spp. and *D. dendriticum* in slaughtered sheep and cattle were lower than the earlier study which carried out in Tehran Province during 2005-2008 [40]. It is likely to be related to the increase of industrial animal husbandry in the province. Most previous researches in parts of Iran showed that the frequency rate of fasciolosis is higher than dicrocoeliosis [23,26], but the results of the present study were different. Our findings are in agreement with the reports of Khanjari *et al*. [40] and Dezfouli and Mokhber [22] in Iran, showing that the frequency rate of dicrocoeliosis is higher than fasciolosis in both sheep and cattle. It seems that the great frequency of dicrocoeliosis is due to the low drug efficacy against *D. dendriticum* in the country. The present data revealed seasonal pattern for *D. dendriticum* in sheep and cattle during winter and spring, respectively. The highest seasonal frequency rate of hydatidosis was seen in spring (Table-2). Seasonal pattern of prevalence and statistical analysis (Chi-square test) showed a significant correlation between hydatidosis, dicrocoeliosis, and fasciolosis prevalence and seasons for sheep in this study (p<0.01). The correlation between hydatidosis and dicrocoeliosis prevalence and season for cattle was also observed for cattle (p<0.01). However, statistical analysis revealed that there is a weak correlation between fasciolosis prevalence and seasons (p>0.01). Tehran, the capital of Iran, imports live farm animals from many regions of the country. Therefore, the discrepancies may be due to the importation of slaughtered animals from different provinces of Iran.

Since hydatidosis and fasciolosis are medically significant parasitic zoonoses in Iran, this survey gives baseline information about livestock, sheep and cattle contamination with hydatid cyst and common liver flukes, and provide important knowledge about epidemiological pattern of parasitic zoonoses in this country.

## Conclusion

A detailed epidemiological study should be conducted to develop animal health, prevention of diseases, and mitigation of economic losses due to the condemnation of infected livers and lungs. Moreover, to reduce these potentially important parasitic infections, standard meat inspection records, safe disposal of infected offal, prevention of access of dogs to raw offal, regular deworming of dogs, and mass chemotherapy of ruminants, to improve the mechanism of slaughterhouses and prohibition of unwarranted killing of animals, to increase the awareness of ranchers, to enhance consciousness of people regarding parasitic infections risks and management practices of animals, and to increase the humans knowledge about not consumption raw watercress and other edible aquatic plants, recommendations are forwarded.

## Authors’ Contributions

MA planned and designed the study, revised the manuscript and done the statistical analysis. The data were collected in the fields by HA. AP drafted the manuscript. All authors read and approved the final manuscript.
